# Interoceptive accuracy is related to the psychological mechanisms of the burning mouth syndrome: a cross-sectional study

**DOI:** 10.1186/s12903-022-02316-w

**Published:** 2022-07-19

**Authors:** Atsuo Yoshino, Naofumi Otsuru, Mitsuru Doi, Toru Maekawa, Takafumi Sasaoka, Shigeto Yamawaki

**Affiliations:** 1 Center for Brain, Mind and KANSEI Sciences Research, Hiroshima University, 1-2-3 Kasumi, Minami-Ku, Hiroshima, 734-8551 Japan; 2 Health Service Center, Hiroshima University, 1-7-1 Kagamiyama, Higashi-Hiroshima, 739-8514 Japan; 3 Department of Physical Therapy, Niigata University of Health and Welfare, 1398 Shimamichou, Kita-Ku, Niigata, 950-3198 Japan; 4 Department of Dental Anesthesiology, Hiroshima University, 1-2-3 Kasumi, Minami-Ku, Hiroshima, 734-8551 Japan

**Keywords:** Chronic pain, Burning mouth syndrome, Interoception, Depression, Anxiety

## Abstract

**Background:**

Different perspectives are needed to understand the pathophysiology of burning mouth syndrome (BMS), including physiological and psychological standpoints. The significance of interoception in chronic pain has been suggested. However, few studies have investigated this relationship in BMS. Therefore, we examined the role of interoception in BMS.

**Methods:**

This is a cross-sectional study. BMS patients (N = 64) participated in the study. We used interoceptive accuracy (IAc) based on the heartbeat counting task. Then, participants were divided into high and low IAc groups, and their scores on clinical assessment including pain and psychological evaluation were compared.

**Results:**

The Visual Analogue Scale scores indicating pain in low IAc patients, but not high IAc patients, were positively correlated with the Beck Depression Inventory-Second Edition (BDI-II) and the State-Trait Anxiety Inventory-State (STAI-S) Scores.

**Conclusions:**

Interoception might play a role in the pathophysiology of BMS.

## Background

International Headache Society’s (IHS) criteria for the diagnosis of primary BMS includes constant intraoral pain with a burning quality, the normal appearance of the oral mucosa, and exclusion of any local or systemic diseases [1]. The pathophysiology of primary BMS might involve abnormal peripheral tongue tissue nerves due to physiological causes such as reduced epithelial density of small fiber-endings [2]. Recent evidence from neurophysiological, neuropathological and brain imaging studies support the understanding neuropathic mechanisms of primary BMS [3]. However, clinical studies have also presented convincing evidence of psychological involvement in the etiology of primary BMS [4, 5]. Many BMS patients frequently have depression and anxiety as comorbid conditions [6]. We have reported that negative emotions increase the intensity of sensory perception in BMS patients during intraoral tactile stimulation [7]. These findings suggest that many different perspectives are needed to understand the pathophysiology of the intraoral somatosensory system in primary BMS.

Interoception is defined as the sensations of internal bodily signals such as heartbeat, breath, and thirst [8]. Emerging behavioral evidence suggests that patients with chronic pain have altered interoception [8] and has shown that chronic pain subjects exhibit lower interoceptive accuracy than healthy controls [8]. Moreover, clinical and biological studies indicate this interoception abnormalities increase somatic complaints and psychological conditions such as depression [8, 9]. Therefore, interoception might play a significant role in chronic pain development. However, to our knowledge, few studies have been conducted on interoception in BMS patients. Therefore, we examined how interoception affects pain intensity in BMS patients using questionnaires assessing the psychological status and heartbeat counting task (HCT). HCT can be used to measure interoceptive accuracy (IAc), representing the general interoceptive capacity. Several studies have sorted participants into high and low interoception perceivers based on the HCT score and compared group differences [10, 11]. We used the identical methodology in this study.

Di Lernia et al. [8] have reported that chronic pain patients with low interoception have difficulties in correctly distinguishing pain-related affective and physical factors. Such patients also have an inability to attribute physical reactions induced by emotional stress to their origin in emotions, and misidentify normal pain perception as more severe and catastrophic experiences, increasing their emotional load [8]. Based on these findings, we hypothesized that there would be a positive association between psychiatric symptoms, including depression and anxiety, and pain experiences in BMS patients with low interoception.

## Methods

### Participants

The participants in this cross-sectional study were 64 female Japanese BMS patients recruited from a dental anesthesia outpatient department of Hiroshima University Hospital. The diagnosis of primary BMS was made according to the classification of IHS by a trained dental anesthesiologist (author M. D.) with more than 15 years of experience [12]. We clinically examined the oral cavity of all the BMS patients, and confirmed that their oral mucosa appeared normal. The exclusion criteria for BMS included known causes of oral burning-like pain, such as vitamin B12 deficiency, diabetes, anemia, thyroid disease, established neurological diseases (e.g., Parkinson's disease), a history of surgery, radiation to the head and neck region, or candidiasis. We also excluded participants with any current psychiatric comorbidity. All the participants gave their written informed consent before participating in this study. Full informed consent, clinical assessment and HCT were performed in the same quiet room in a dental anesthesia outpatient department of Hiroshima University Hospital. We decided the minimum number of participants based on previous studies on the association between pain and psychiatric symptoms such as depression and anxiety of chronic pain patients [13–15]. Previous studies have shown that the measured effect sizes of association between pain and psychiatric symptoms were 0.40 [13], 0.58 [14], and 0.42 [15], with a mean effect size of 0.47. We determined that the statistical test would need a minimum of 30 participants to detect this association with the power of 0.80 and α of 0.05. The study was conducted according to the protocol approved by the Ethics Committee of Hiroshima University.

### Clinical assessment

#### Pain evaluation

The Visual Analogue Scale (VAS) was used as a self-report measure for assessing pain intensity in daily life. Participants rated their mean pain intensity for one week when they felt pain. We also used the Pain Catastrophizing Scale (PCS) [16], a 13-item self-report inventory designed to assess the extent to which a person engages in catastrophic thinking in response to pain stimuli.

#### Psychological evaluation

The participants completed the Beck Depression Inventory-Second Edition (BDI-II) [17], which is a widely used 21-item self-report measure of depressive symptom severity, demonstrating good psychometric properties [17]. They also completed the State-Trait Anxiety Inventory (STAI) [18]. The STAI is a self-report questionnaire that includes 40 items, which has two scales for differentiating state anxiety (STAI-S) related to transitory or situational states, and trait anxiety (STAI-T) related to consistently more stable characteristics of an individual that resemble a personality trait [18].

#### IAc assessment

We used the HCT described by Schandry to assess IAc [19]. Participants focus on their heartbeats in the HCT and count them silently for a specific period (25, 35, 45, and 60 s) without using any alternative methods for detecting the heartbeat. In this study, the instructor indicated the beginning and end of each counting interval using a start and stop tone. The counting intervals were separated by 30 s rest periods. We used an ECG monitor to measure heartbeats objectively. IAc scores were calculated to indicate the participant's heartbeat perception accuracy by using the following equation.

$${\text{IAc}} = 1 - \frac{{1}}{{4}}\sum (\frac{{\left| {\text{recorded heartbeats}} - {\text{counted heart beats}} \right|}}{{\text{recorded heartbeats}}})$$

### Data analysis

We conducted two-sample *t*-tests to assess the differences in VAS, BDI-II, STAI, and PCS scores between the two groups. The threshold for statistical significance was set at *p* < 0.05. We also conducted Pearson correlation analysis between VAS, IAc, and psychological variables, including PCS, BDI-II, and STAI (STAI-S and STAI-T), to assess group’ associations between these scores. The Bonferroni correction indicated *p* < 0.05/10 = 0.005. All the data were analyzed using SPSS for Windows 21.0 (SPSS, Chicago, IL, USA).

## Results

### Participant’s characteristics

Table 1 shows the detailed demographic and clinical characteristics of the participants. The median value of IAc scores was 0.71 (S.D. = 0.17). We divided the 64 participants into high and low IAc groups using the median split method [11]. The average HCT score of the high IAc group was 0.83 (S.D. = 0.08, n = 32) and the low IAc group 0.56 (S.D. = 0.11, n = 32). The median score in the present study was nearly identical to a previous study [11]. There were no significant differences in any of the items of the demographic and clinical characteristics between high and low IAc groups.

**Table 1:** (a) Demographic and psychometric variables of patients; (b) Pearson correlation coefficients (*r*) for the relationship between pain intensity and psychometric evaluation

(a)				
	High IAc Patients (n = 32)	Low IAc Patients (n = 32)	High IAc vs Low Iac	
	M (SD)	M (SD)	Statistic	*p*
Age	56.9 (11.0)	55.3 (12.5)	*t* (62) = 0.54	0.59
Female	32	32	–	–
VAS	50.0 (25.9)	52.9 (25.1)	*t* (62) = 0.46	0.65
BDI-II	12.9 (6.4)	10.9 (8.0)	*t* (62) = 1.08	0.28
STAI-S	49.7 (7.7)	49.2 (10.1)	*t* (62) = 0.22	0.82
STAI-T	47.8 (11.2)	46.4 (11.5)	*t* (62) = 0.52	0.61
PCS	30.0 (10.1)	29.7 (9.3)	*t* (62) = 0.15	0.88
IAc	0.83 (0.08)	0.56 (0.11)	*t* (62) = 11.19	< 0.001

(b)				
Variables	High IAc Patients	Low IAc Patients		
BDI-II	0.048 (*p* = 0.80)	0.53* (*p* = 0.002)		
STAI-S	0.19 (*p* = 0.30)	0.50* (*p* = 0.004)		
STAI-T	0.01 (*p* = 0.94)	0.36 (*p* = 0.040)		
PCS	0.48 (*p* = 0.006)	0.47 (*p* = 0.007)		
IAc	0.13 (*p* = 0.48)	− 0.13 (*p* = 0.49)		

*VAS* Visual Analogue Scale, *BDI-II* Beck Depression Inventory-Second Edition, *STAI-S* State-Trait Anxiety Inventory-State, *STAI-T* State-Trait Anxiety Inventory-Trait, *PCS* Pain Catastrophizing Scale, *IAc* Interoceptive Accuracy

*p < 0.005

### Correlation analysis

**Fig. 1:**
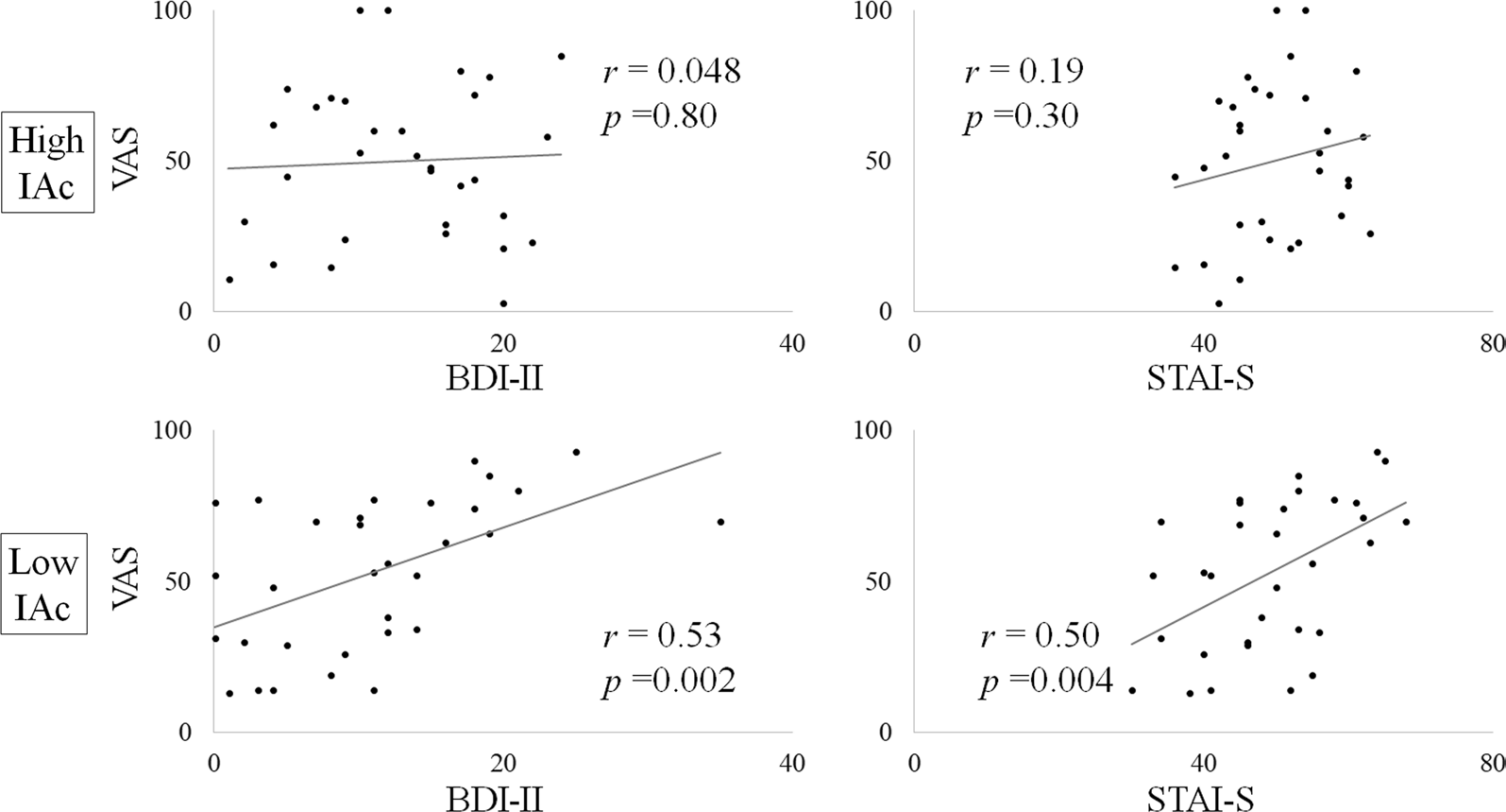
Relationship of VAS with BDI-II and STAI-S. VAS scores were positively correlated with BDI-II and STAI-S in BMS patients with low IAc. VAS: Visual Analogue Scale, BDI-II: Beck Depression Inventory-Second Edition, STAI-S: State-Trait Anxiety Inventory-State, IAc: Interoceptive Accuracy

As shown in Table 1 and Fig. 1, the VAS scores of BMS patients with low IAc were positively correlated with BDI-II (*r* = 0.53) and STAI-S (*r* = 0.50; *p*s < 0.005) scores. Moreover, there was no significant relationship between VAS and any other variable in either groups, although there was a strong association between VAS and PCS scores (high IAc; *r* = 0.48 (*p* = 0.006), low IAc; *r* = 0.47 (*p* = 0.007)).

## Discussion

This study is the first to investigate the role of IAc and psychological evaluation in BMS patients’ pain intensity. The findings indicated that VAS scores of patients with low IAc, but not in patients with high IAc, were positively correlated with BDI-II and STAI-S.

### Role of interoception in BMS

This study found a positive relationship between clinical pain symptoms of low IAc BMS patients and BDI-II or STAI scores. Individuals with low interoception have difficulties in regulating emotions, and it has been suggested that it is important to recognize the bodily states correctly to understand and regulate emotions [20]. Emotion regulation means that one handles emotional responses well through control by involving cognitive mechanisms and physical reactions [21]. Emotion regulation impairments have been implicated as one possible mechanism of enhanced pain perception by patients having negative psychiatric symptoms such as depression [22]. Therefore, we suggest that pain perception would be easily enhanced in BMS patients with low IAc when they have depression or anxiety, because they would not be able to handle emotional response by their disability of emotion regulation, which would contribute to enhanced pain perception as somatic complaints. Schaefer et al. reported improving clinical symptoms in somatoform pain disorders by training patients to improve interoception [23]. We suggest that improving interoception could be an appropriate future treatment option for BMS patients with low IAc. On the other hand, we did not find the specific cause of pain symptoms in high IAc BMS patients. Therefore, more research is needed to clarify the mechanisms of pain symptoms of BMS patients further. It has been reported that pain intensity in BMS patients was affected by various factors such as sleep problems, pain hypervigilance and pain catastrophizing, except depression and anxiety [13, 24]. In this present study, there was a strong association between VAS and PCS scores in each group. Our results may also represent that pain catastrophizing enhances pain intensity in BMS patients as a different mechanism from interoception.

The present study has several limitations. Our exclusion criteria for participants did not include all possible treatment effects that might influence pain perception, interoception and psychiatric symptoms of patients, such as the use of antidepressants. Furthermore, we did not establish a healthy control group, and therefore we could not show the general association between psychiatric symptoms and interoception, although we could clarify the role of interoception within BMS patients. Finally, BMS patients show general time-dependent changes of symptoms, such as more severe pain in the evening [3]. However, this study just analyzed the participants’ mean pain intensity for one week. Therefore, further studies are needed to examine how pain intensity changes during the day are correlated with depression and anxiety in high and low IAc groups.

## Conclusions

This study found that the pain intensity felt by BMS patients with low interoception was positively associated with depression and anxiety. Therefore, we suggest that research on interoception might contribute to clarifying the pathophysiology of BMS. We expect that improving interoception would become a future treatment option for BMS due to similar research.

## Data Availability

The datasets used and/or analyzed during the current study are available from the corresponding author on reasonable request.

## Abbreviations

BDI-II: Beck Depression Inventory-Second Edition
BMS: Burning mouth syndrome
ECG: Electrocardiogram
HCT: Heartbeat counting task
IAc: Interoceptive accuracy
IHS: International Headache Society
PCS: Pain Catastrophizing Scale
S.D.: Standard deviation
STAI-S: State-Trait Anxiety Inventory-State
STAI-T: State-Trait Anxiety Inventory-Trait
VAS: Visual Analogue Scale
